# Skeletal Muscle Phenotypically Converts and Selectively Inhibits Metastatic Cells in Mice

**DOI:** 10.1371/journal.pone.0009299

**Published:** 2010-02-18

**Authors:** Ara Parlakian, Iman Gomaa, Sounkary Solly, Ludovic Arandel, Alka Mahale, Gustav Born, Giovanna Marazzi, David Sassoon

**Affiliations:** 1 Myology Group, UMR S 787 Inserm, Université Paris VI/Pierre et Marie Curie, Paris, France; 2 William Harvey Research Institute, University of London, London, United Kingdom; Department of Oncological Sciences, Mount Sinai School of Medicine, New York, New York, United States of America; Roswell Park Cancer Institute, United States of America

## Abstract

Skeletal muscle is rarely a site of malignant metastasis; the molecular and cellular basis for this rarity is not understood. We report that myogenic cells exert pronounced effects upon co-culture with metastatic melanoma (B16-F10) or carcinoma (LLC1) cells including conversion to the myogenic lineage *in vitro* and *in vivo*, as well as inhibition of melanin production in melanoma cells coupled with cytotoxic and cytostatic effects. No effect is seen with non-tumorigenic cells. Tumor suppression assays reveal that the muscle-mediated tumor suppressor effects do not generate resistant clones but function through the down-regulation of the transcription factor MiTF, a master regulator of melanocyte development and a melanoma oncogene. Our findings point to skeletal muscle as a source of therapeutic agents in the treatment of metastatic cancers.

## Introduction

Understanding the cellular and molecular mechanisms leading to metastasis is of key importance for targeting metastatic cells since no efficient method to block metastasis exists [1], [2], [3], [4]. The metastatic conversion of a tumor cell involves cell-autonomous changes which alter cellular cross-talk in the tissue environment. Interactions between tumor cells and the host organ rely on similar mechanisms governing interactions during embryogenesis [5], [6], [7]. The host organ microenvironment is critical in regulating metastatic tumor growth [4], [8], [9], [10]. Most tissues are subject to secondary tumor growth with the striking exception of skeletal muscle in which malignant metastases are very rare [11], [12], [13], [14]. This is particularly surprising because skeletal muscle comprises ∼40 percent of total body mass and is highly vascularized [11], [13], [14], [15]. Willis and colleagues reported a frequency of 0.8% of intramuscular metastasis in autopsied cases [16]. A retrospective study over a 30-year period revealed that only 1.6% of soft tissue sarcomas (muscle) examined was of metastatic origin [12]. The origins of these metastases were mostly primary lung carcinomas (70%) and malignant melanomas (17%) [12]. The rarity of malignant metastases in skeletal muscle led Paget to propose the “seed and soil hypothesis” over 100 years ago, namely that metastasis is a function not only of the type of tumor (“seed”) but also of the host tissue (“soil”) [17].

The cellular and molecular mechanisms underlying the rarity of secondary metastasis in skeletal muscle have remained elusive. Several studies have linked muscle activity with inhibition of tumor growth [18], [19], [20]. The influence of β-adrenergic receptors on muscle vasculature and blood flow may pose a limiting factor for metastatic tumor growth [21]. Angiogenesis is required for all tumor growth [22]. Production of lactic acid by tumor cells promotes angiogenesis through activation of vascular endothelial growth factor (VEGF) [23], [24]. Skeletal muscle produces high levels of lactic acid suggesting that muscle vasculature blood may not respond to additional lactic acid secretion by invasive tumor cells [25]. Over-production of lactic acid is detrimental for tumor development [26]. Several groups have shown that muscle conditioned media exert a cytostatic effect upon tumor cells [27], [28]. Adenosine and other low molecular weight factors were suggested to be responsible for the inhibition of tumor cell growth through the A3 adenosine receptor, however, adenosine depleted conditioned media still retained a cytostatic effect [29], [30].

In this study, we make use of the highly metastatic murine melanoma B16F-10 [31], [32] as well as the Lewis Lung carcinoma [33] cell lines, within the context of a skeletal muscle microenvironment *in vitro* and *in vivo*. In man, malignant melanoma accounts for only 4% of all dermatologic cancers but is responsible for 80% of skin cancer mortality because of its high metastatic rate (American cancer society, 2003). Several molecules implicated in melanoma tumorogenesis have been identified [34]. Polymorphisms in MC1R, a gene involved in melanin production, increase the risk of melanoma following exposure to UV light [35]. In melanoma patients, mutations, deletions and genomic amplifications of genes involved in cell cycle control, apoptotic pathways, and in oncogenes such as CDKN2A, PTEN, AKT, N-RAS, and BRAF occur at different rates [36], [37], [38], [39]. Altered expression of adhesion molecules such as cadherins, integrins, and the WNTs are implicated in the metastatic spreading of melanoma [40], [41]. Finally, MiTF, a key transcription factor in survival, maintenance and differentiation of melanocytes is detected in most malignant melanomas and the gene is amplified in 10 to 20% of cases of metastasis [42], [43], [44], [45].

In this study, we show that interactions between melanoma cells with either primary or established myogenic cell lines lead to specific changes in melanoma cell behavior, including inhibition of melanin pigment production. In addition, we observe that GFP labeled melanoma and Luis Lung carcinoma cells fuse to form myotubes when co-cultured with myoblasts. Injection of GFP-labeled melanoma cells into uninjured or regenerating skeletal muscle results in GFP positive myofibers indicating that these cells are recruited into muscle fibers. Serum free muscle-conditioned media exerts a cytostatic and cytotoxic effect upon metastatic cells but has little effect upon non-metastatic cells suggesting that muscle secreted factors are not general cell growth inhibitors. Surprisingly, tumor suppression assays fail to generate ‘muscle-resistant’ melanoma tumor cells such that cells subject to multiple rounds of selection remain sensitive to the growth suppression effects exerted by muscle, and is accompanied by down-regulation of the transcription factor MiTF. Furthermore, MiTF downregulation is dependent upon cell contact and myogenic cell density in the co-culture. These findings suggest a mechanism whereby skeletal muscle inhibits secondary metastasis by the secretion of factors as well as by cell-to-cell interactions that hijack metastatic cells, leading to myogenic conversion and reduced growth by acting through signaling mechanisms not normally involved in tumor suppression.

## Results

### Metastatic Melanoma Cells Fail to Invade and Colonize Skeletal Muscle

We injected 5×10^5^ B16-F10 melanoma cells into the tail vein or the peritoneum of C57/Bl6 syngeneic mice and analyzed for the presence of tumors 3 weeks after injection. The B16-F10 melanoma cell line produces melanin-expressing tumors allowing for easy identification of tumors in dissected tissues. Three weeks following tail vein injection of B16-F10 cells, we observed the presence of black pigmented growths in all organs examined including lung, liver, spleen, intestine, and heart (Table 1). In contrast, no metastases were observed in skeletal muscles of the limbs and diaphragm, which were examined in detail (Table 1). We observed that if cells were injected intraperitoneally (IP) versus the tail vein, tumor frequency shifted among tissues (i.e. all animals showed intestinal tumors following IP injections versus 4 out of 12 following tail vein injections) (Table 1). Nonetheless, regardless of route of injection, no tumors were ever observed in skeletal muscle tissues.

**Table 1:** Distribution of B16 melanoma tumour cells in mouse organs following different delivery routes.

Injection route	N°	Lung	Liver	Spleen	Bone	Heart	Skeletal muscle		Intestine
							TA	Other muscles^c^	
**iv Tail** (B16*F10*)	12	12	5	6	0	9^a^	0	0	4
**Ip** (B16*F10*)	6	0	1	1	0	0	0	0	6
**iv Tail** (B16*F0*)	4	4	2	0	3	4^b^	0^d^	0	1

a Tumors in the endocardium 6/9; tumors in the myocardium 3/9.

b Tumors in the endocardium 3/4; tumors in the myocardium 1/4.

c Fore and hind limb muscles + diaphragm were examined in detail.

d Few isolated melanin-secreting B16-F0 cells were found associated with muscle surface of a single individual. No evidence of tumor invasion was observed.

iv: intra-venous 5×10^5^ cells injected.

ip: intraperitoneal 1×10^3^ cells injected.

TA: *Tibialis anterior*.

Previous studies have demonstrated that the B16-F10 cell line displays preferential distribution to the lung following tail vein injection [46], [47], therefore we tested the B16-F0 subclone that has a wider metastatic range of tissues and does not preferentially invade the lung [32]. Three weeks following tail vein injection of 5×10^5^ B16-F0, we observed tumors in the lung, liver, intestine, heart, and bones (Table 1). As for the F10 cell line, no tumors were detected in skeletal muscle despite their presence in the adjacent bones. Only few isolated melanin-secreting B16-F0 cells were detected in the epimysium closely associated with the *Tibialis Anterior* (TA) muscle of one animal, consistent with the low incidence of skeletal muscle metastatic tumor invasion and growth.

### Myogenic Cells Inhibit B16-F10 Melanoma Cells Differentiation *In Vitro*

**Figure 1:**
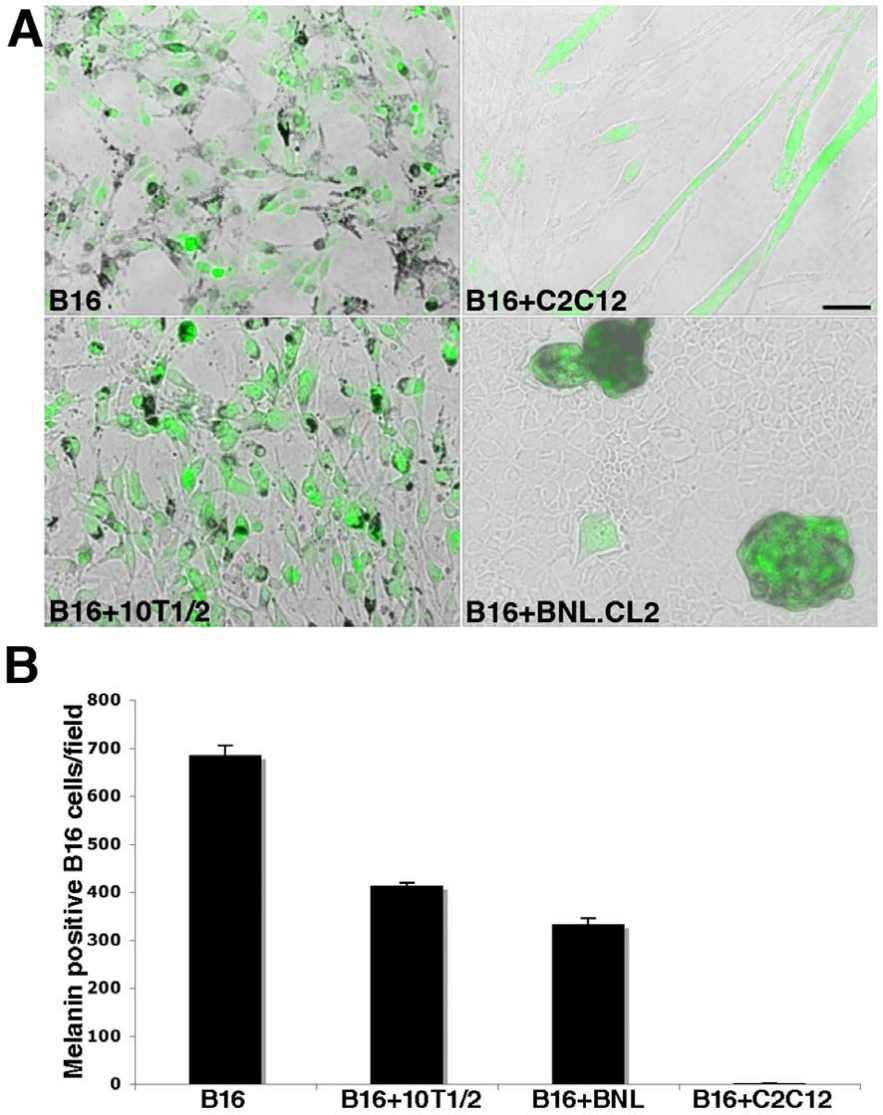
Myogenic cells inhibit B16-F10 melanoma cells differentiation *in vitro.* A) Photomicrographs of GFP expressing melanoma cells (B16) grown alone or in co-culture with myogenic cells (C2C12), fibroblasts (10T1/2), or liver cells (BNL.CL2). We note an inhibition of melanin production in B16 co-cultured with C2C12 as well as a change in their morphology. Scale bar  = 50 µm. B) Quantification of the number of melanin secreting B16 cells grown alone, or in co-culture with fibroblasts, liver cells, or myogenic cells reveals that myogenic cells completely abolish melanin production in B16 cells.

To follow melanoma tumor behavior at the cellular level, we generated GFP expressing B16-F10 cells (B16-GFP) and challenged them directly in culture with a variety of cell types including skeletal muscle cells. We co-cultured B16-GFP cells over a range of cell ratios ranging from 1∶1 to 1∶400 with fibroblasts (10T1/2), liver cells (BNL.CL2), and murine skeletal muscle (C2C12) cell lines. Due to the inherently high proliferative rate of melanoma cells, the optimal ratio of tumor cells to other cell lines was determined to be 1∶100 to allow for proper visualization of the two cell types at the end of the experiment. Once co-cultured cells were confluent, serum levels were decreased to induce myogenic differentiation (Differentiation Media, DM). We note that even though DM was used to provoke myogenic differentiation, all combinations of cell types were subjected to the identical treatment allowing for a direct comparison of outcomes. Following 4–6 days in DM, B16-GFP differentiate into melanin producing cells. When B16-GFP are co-cultured with fibroblasts (10T1/2), we observe that the two cell types mix evenly (Figure 1A) and B16-GFP cells retain their ability to secrete black melanin pigments (Figure 1A,B). When co-cultured with liver cells (BNL.CL2), the B16-GFP melanoma cells segregate presumably due to different cellular adhesion properties, forming islands of GFP+ cells expressing melanin distributed among the BNL.CL2 liver cells (Figure 1A). In contrast, when B16-GFP cells are co-cultured with myogenic cells (C2C12), they remain closely associated with differentiating myogenic cells, and acquire an elongated morphology (Figure 1A). In addition to this striking change in cell shape, we observed that none of the B16-GFP cells secrete melanin (Figure 1A). A similar outcome was observed when melanoma cells and myogenic cells where co-cultured at a ratio of 1∶1 (data not shown). We note that B16-GFP cells are present at a lower number following co-culture with myoblasts as compared to co-cultures with other cell types (Figure 1A) suggesting either a lower rate of proliferation and/or increased cell death. Quantification of the number of melanin secreting cells in the different co-culture conditions reveals an absence of melanin positive cells (1.86 cells ±0.35/field) in co-cultures with myogenic C2C12, while in fibroblast and liver cell co-cultures, we observe a mean value of 413±6 and 333±12.96 melanin positive cells/field respectively (Figure 1B). We conclude that skeletal muscle cells inhibit melanoma cell differentiation (measured by melanin expression).

### Metastatic Cancer Cells Cultured with Skeletal Muscle Cells Participate to the Myogenic Program

**Figure 2:**
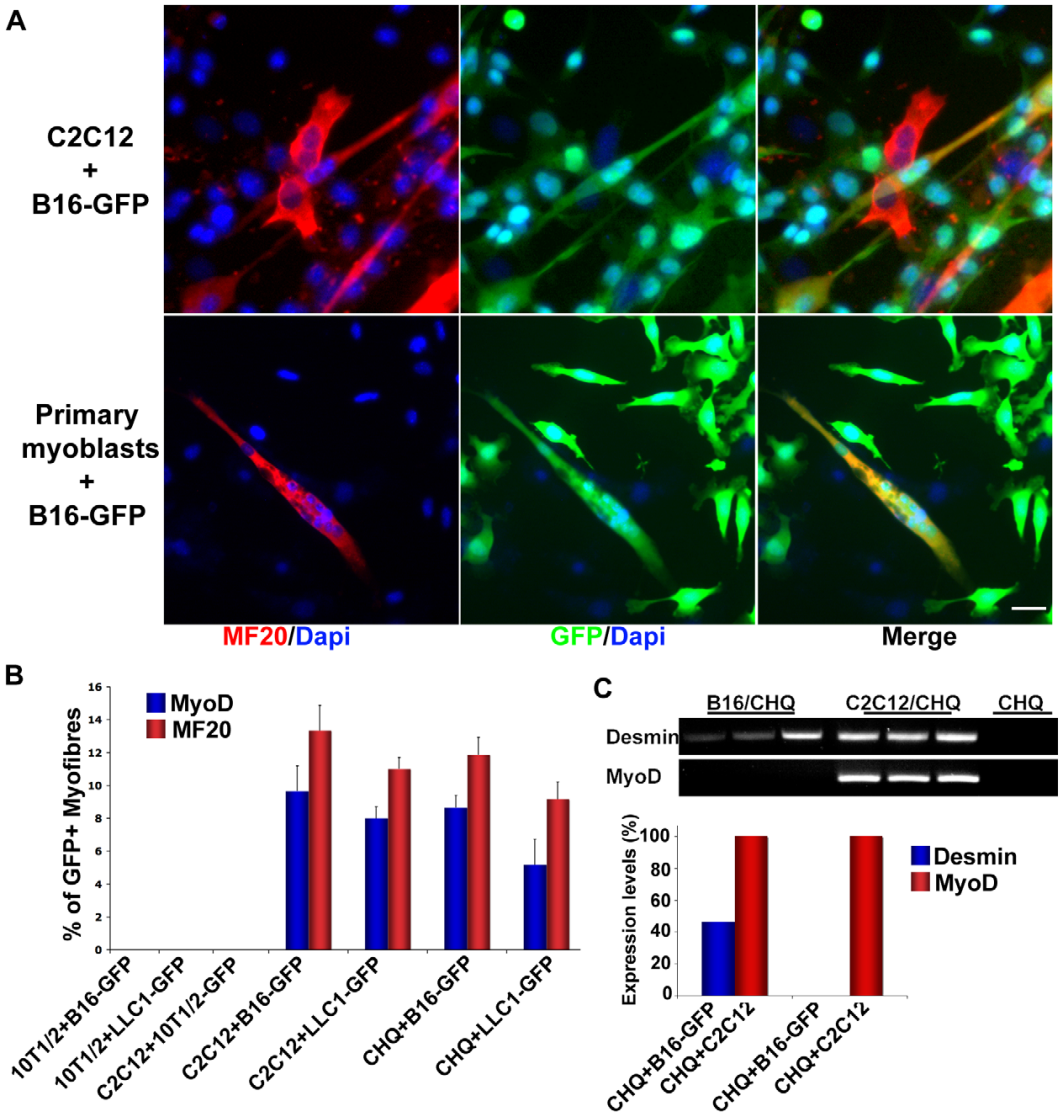
Murine and human muscle cells recruit tumor cells in the myogenic program. A) Representative photomicrographs of GFP expressing B16 cells (green) grown and differentiated in co-culture with either C2C12 mouse myogenic cell line or mouse primary myoblasts and stained for myosin heavy chain (MF20, red). GFP labeled melanoma cells fuse with the C2C12 cells forming chimeric green myotubes, which are positive for MF20. Nuclei were visualized by DAPI staining (blue). Scale bar  = 20 µm. B) Quantification of GFP expressing myotubes positive for either MyoD or Myosin heavy chain (MF20), from co-cultures of GFP-labeled melanoma cells (B16-GFP) or GFP-labeled Luis Lung Carcinoma cells (LLC1-GFP) with mouse myogenic cells (C2C12), human primary myogenic cells (CHQ), or fibroblasts (10T1/2) immunostained as shown in panel A. Only murine and human muscle cells can recruit Luis Lung Carcinoma (LLC1-GFP) and melanoma(B16-GFP) cells into the myogenic program. This phenotypic conversion is specific to cancer cells and does not occur in co-cultures of GFP labeled fibroblasts with myogenic C2C12 cells. Values represent the mean percentage of at least 3 independent experiments ± SEM. C) Expression of murine Desmin and MyoD transcripts in co-cultures of B16-GFP/CHQ; C2C12/CHQ and CHQ cells alone(RT-PCR). The graph shows the expression levels of Desmin and MyoD transcripts in co-cultures relative to the expression in C2C12/CHQ co-cultures. Experiments were performed in triplicates.

The elongated morphology observed in melanoma cells co-cultured with myogenic cells suggested that B16 cells “fuse with myogenic cells and might” undergo myogenic conversion. We therefore examined the expression of skeletal muscle specific markers (MyoD and Myosin heavy chain (MF20)) in co-cultures of B16-GFP cells with either C2C12, or primary murine myoblasts using immunofluorescence. As shown in Figure 2, we can identify myotubes (multinucleated cells) positive for GFP and MF20, indicating that GFP melanoma cells have fused with the differentiating myogenic cells. About 13% of the total number of myotubes in co-cultures of GFP-labeled melanoma cells and C2C12 cells are positive for MF20 and GFP (Figures 2A, 2B). In addition, MyoD, a muscle transcription factor expressed in myoblasts and early myotubes, was detected in GFP positive myotubes (9.6% of the total number of myotubes) (Figure 2B). This fusion process was specific to melanoma cells and did not occur in co-cultures of GFP labeled fibroblasts with myogenic C2C12 cells (Figure 2B). To test whether the ability of myogenic cells to fuse with metastatic cells was limited to melanoma, we extended our analyses to the Luis Lung carcinoma line [33]. We therefore generated GFP expressing Luis Lung Carcinoma cells (LLC1-GFP) and tested them in co-culture with myoblasts. As seen for the GFP-B16 cells, the LLC1-GFP cells acquired an elongated morphology and ultimately fused with C2C12 cells giving rise to chimeric green myotubes (Figure S1) that express MyoD (8%) and MF20 (11%) (Figure 2B). No MyoD nor MF20 expression was detected in co-cultures of B16-GFP or LLC1-GFP with 10T1/2 fibroblasts (Figure 2B). Lastly, B16-GFP and LLC1-GFP cells fuse with human primary myoblasts (CHQ) giving rise to chimeric green myotubes that express MyoD (5%) and MF20 (9.1%) (Figure 2B). We explored a number of different conditions including testing whether B16-GFP cells fuse with C2C12 cells previously differentiated into myotubes. As observed in coculture with myoblasts, B16-GFP cells plated onto fully differentiated myotubes fuse with myotubes and do not secrete melanin (data not shown). In order to determine if the B16-GFP melanoma cells are reprogrammed and convert to the myogenic lineage upon fusion, we co-cultured murine B16-GFP melanoma cells with human primary myoblasts (CHQ) in a 1/500 ratio. Using RT-PCR, we searched for the presence of murine muscle specific transcripts and detected the presence of murine Desmin (an early myogenic marker) suggesting the phenotypic conversion of the melanoma cells into a myogenic lineage upon fusion (Figure 2C). No murine MyoD transcripts were detected indicating that the conversion is probably driven by myogenic transcription factors from neighboring human nuclei within the same cytoplasm. Semi-quantitative analysis revealed that the expression levels of murine Desmin reached an average of 46%±12.9 in B16-GFP/CHQ co-cultures as compared to murine C2C12/CHQ co-cultures (Figure 2C). No detectable levels of murine Desmin transcripts were present in 10T1/2 fibroblasts/CHQ co-cultures (not shown). Finally, the absence of murine transcripts (Desmin and MyoD) in CHQ cultures alone indicates the specificity of the primers to amplify only murine transcripts (Figure 2C).

### B16-GFP Melanoma Cells Undergo Myogenic Conversion *In Vivo*

**Figure 3:**
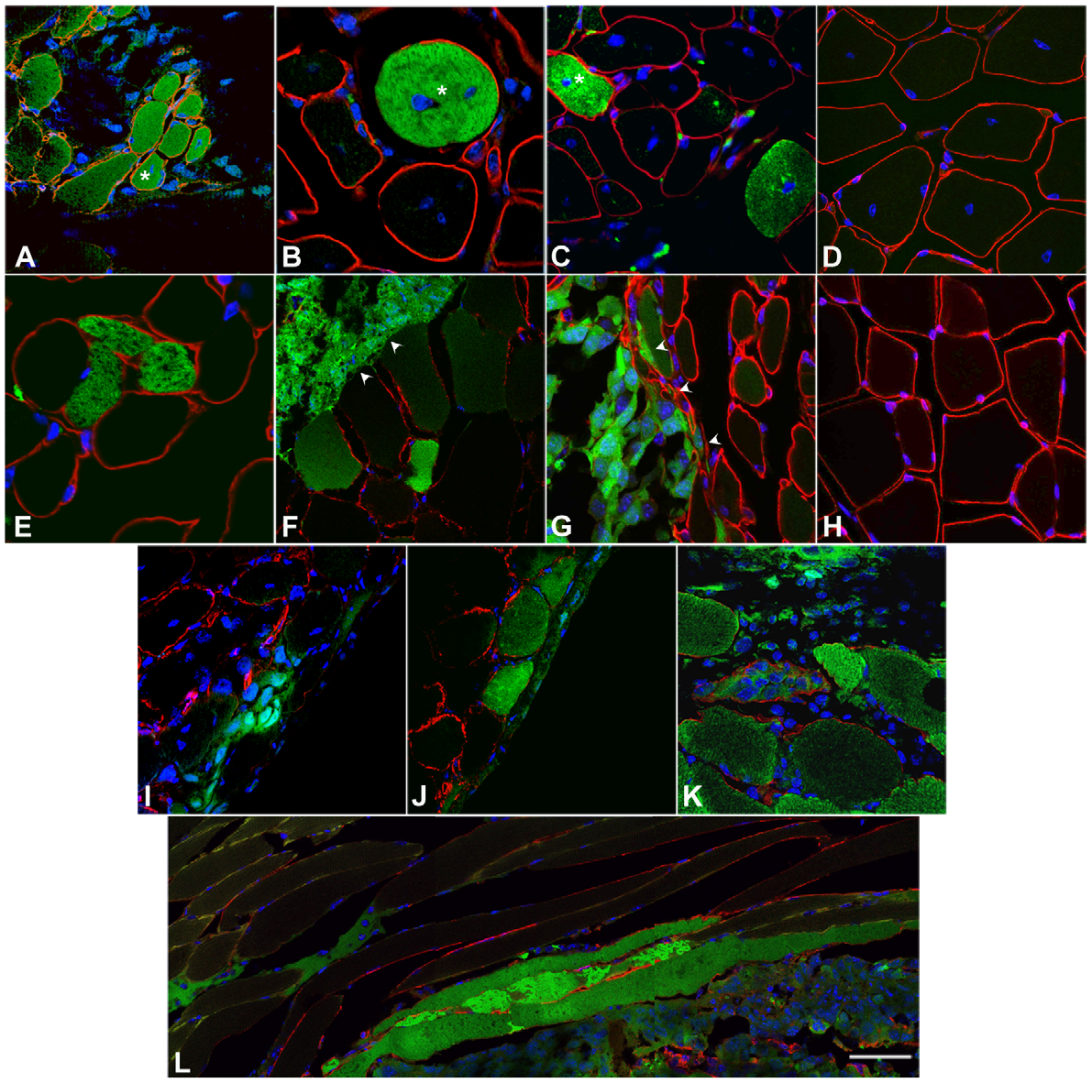
B16 melanoma cells fuse with muscle fibers in vivo. A-H) Confocal micrographs of representative cross sections of TA muscles, showing fusion of GFP-labeled B16 melanoma cells with injured (A-C) and non-injured (F-H) muscles, 2 weeks after intramuscular injection. The basal lamina is identified by the red laminin immunostaining. Nuclei are visualized by DAPI staining (blue). We note GFP+ myofibers with centrally located nuclei (A-C asterisk), characteristic of muscle fibers undergoing regeneration. Green, GFP+ myofibers are observed in non-regenerating muscle as well, indicating that GFP+ melanoma cells fuse to pre-existing fibers. In both cases, we note GFP positive fibers within the site of injection (A), adjacent to the tumor (F,G), as well as in areas devoid of tumor (B,C,E). At the periphery of the tumor we note a well defined boundary between the tumor and the muscle tissue (arrowheads). No green fibers were detectable in the controlateral control injured and non-injured muscles injected with PBS (D,H). I-K) Representative cross sections of TA muscles, 2 weeks after intrafemoral artery injection of GFP-labeled B16 melanoma cells. L) Confocal micrographs of a representative longitudinal section of TA muscle, showing homogenous expression of GFP along the muscle fiber length, 2 weeks after intramuscular injection of GFP-labeled B16 melanoma cells. Scale bar  = 20 µm.

To determine whether melanoma cells display a similar myogenic conversion in vivo, we injected 1×10^3^ B16-GFP cells into injured and non-injured Tibialis Anterior muscles of C57/Bl6 syngeneic mice. Mice were sacrificed at 2 and 3 weeks following B16-GFP injection and selected muscles (soleus, gastrocnemius, and tibialis anterior) from injected legs as well as lung, bone, spleen, liver, heart were collected, analyzed for the presence of tumors, and frozen for immunofluorescence analysis. Three weeks following B16-GFP injection, we detected small melanoma nodules that displayed well-defined boundaries and did not invade the surrounding muscles such as the gastrocnemius and the soleus (data not shown). No melanoma cells were detected in any of the organs examined (lung, bone, spleen, liver and heart). To identify melanoma cell fate, we analyzed injured and non-injured muscles 2 weeks following injection of B16-GFP cells. Muscle tissue is composed of multinucleate myofibers surrounded by a basal lamina (extra-cellular matrix), which delineate the fibers from the interstitium consisting of vessels and connective tissue [48]. In the case of injured muscle, newly regenerated myofibers can be recognized by the presence of centrally located myonuclei [49]. Frozen sections were immunostained for laminin to visualize the basal lamina of the fibers. Following injection of B16-GFP melanoma cells, GFP+ myofibers were detected in both injured (Figure 3A-C) and non-injured (Figure 3E-G) muscles. In most cases, the GFP+ fibers are in close proximity with the melanoma cells (Figure 3). The basal lamina of these fibers often appeared irregular, suggesting that the melanoma cells disrupt the extra-cellular matrix of the myofiber or that neighboring muscle cells respond to the presence of melanoma cells and alter their ECM composition (Figure 3). Consistent with our macroscopic observations, there was a tightly defined boundary at the tumor-muscle border (Figure 3F,G arrowheads). In non-injured muscles, the GFP+ myofibers have peripheral nuclei (unlike muscle fibers undergoing regeneration), revealing that GFP+ melanoma cells fuse to pre-existing fibers and that regeneration is not a prerequisite condition for tumor cells participation into myofibers. No green fibers were observed in controlateral control injured and non-injured muscles (Figure 3D and 3H), which received an injection of PBS (see Materials and Methods).

To assess whether metastatic cells can cross the vessel barrier into muscle tissue in vivo, 1×10^5^ B16-GFP cells were injected into the femoral artery of C57/Bl6 syngeneic mice. To prevent significant circulation of cells to other organs, the femoral artery was clamped during and just after the injections as described in Material and Methods. Two weeks after intrafemoral injection, we note GFP+ myofibers next to melanoma in both uninjured (Figure 3J) and injured muscle (Figure 3K), as seen with the intramuscular injections as well as cells under the the muscle fascia (Figure 3I). Longitudinal sections of intramuscularly injected TA muscles show that GFP is homogeneously distributed along the length of the muscle fiber (Figure 3L). Taken together, these data suggest that tumor cells are able to cross the vessel barrier and that skeletal muscle is able to “recruit” melanoma cells into the myogenic program and exert an inhibitory effect on tumor growth and invasion in vivo.

### Conditioned Media from C2C12 Myogenic Cells Exert an Apoptotic Effect on Tumorigenic Cells

**Figure 4:**
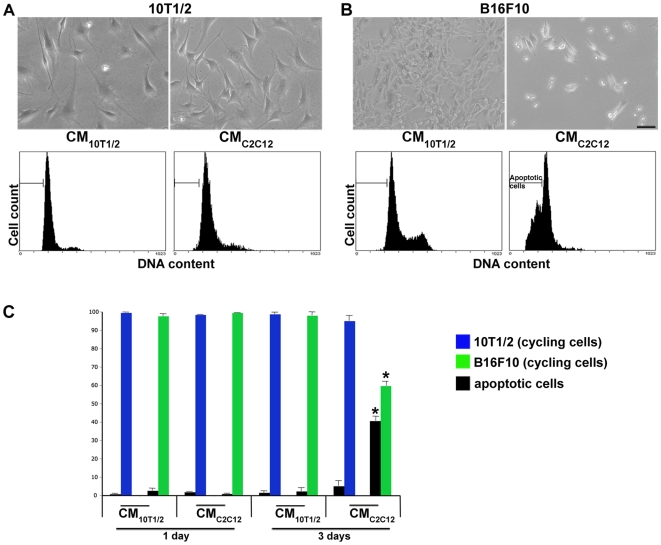
Apoptotic effect of the conditioned media from C2C12 muscle cells on B16 melanoma cells. A,B, upper panels) Phase contrast photomicrographs of 10T1/2 fibroblasts (A) and B16-F10 melanoma cells (B) cultured in serum free conditioned media from 10T1/2 fibroblasts (CM_10T1/2_) or in serum free conditioned media from muscle cells (CM_C2C12_) for 3 days. After 3 days in CM_C2C12_, B16 melanoma cells are less numerous and appear rounded and refractive (B). Scale bar  = 50 µm. A,B, lower panels) Propidium iodide flow cytometry cell cycle analysis of 10T1/2 fibroblasts (A) and B16 melanoma cells (B) in the different culture media. The bars indicate the fraction of apoptotic cells for each experimental condition. The cell cycle profile is notably changed in melanoma cells incubated for 3 days in muscle cells conditioned media. C) Percentage of apoptotic versus cycling cells of 10T1/2 fibroblasts (blue bars) and B16-F10 melanoma cells (green bars) cultured in either serum free conditioned media from 10T1/2 fibroblast (CM_10T1/2_) or C2C12 muscle cells (CM_C2C12_) after 1 and 3 days post incubation (dpi). Only B16F10 melanoma cells exhibit a significantly high level of apoptotic cells (40.5%±2.5) when cultured in CM_C2C12_ for 3 days (p≤0.05). Values represent the mean % ± SEM of 3 independent experiments.

Co-culture experiments with B16-GFP or LLC1-GFP and differentiating C2C12 cells gave rise to cultures which appeared to contain less tumor cells (Figure 1A and Figure S1). It is well accepted that acquired resistance to apoptotic and/or growth arrest signals plays a major role in tumor progression [50], [51]. To determine if diffusible factors secreted by muscle cells induce cell cycle arrest and/or cell death in B16 cells, we collected serum-free conditioned media from C2C12 muscle cultures (CM_C2C12_) and from 10T1/2 fibroblast cultures (CM_10T1/2_). We tested conditioned media on B16 cells as well as C2C12 myoblasts and 10T1/2 fibroblasts and monitored cell morphology, number, and cell cycle status using propidium iodide uptake followed by FACs analysis. 10T1/2 fibroblasts grown in CM_10T1/2_ or CM_C2C12_ display robust growth and do not display overt changes in morphology (Figure 4A, upper panels). Furthermore, we did not observe significant differences in cell cycle profiles of 10T1/2 fibroblasts exposed to CM_10T1/2_ or CM_C2C12_ (Figure 4A, lower panels). After one day in fibroblasts conditioned media (CM_10T1/2_) 0,6% of the 10T1/2 fibroblasts were apoptotic versus 2,5% of apoptotic cells in cultures incubated in conditioned media from myogenic cells (CM_C2C12_) (Figure 4C). The percentage of apoptotic fibroblast cells did not vary 3 days after incubation in both conditioned media (Figure 4C). When B16 melanoma cells were incubated for 1 day in CM_10T1/2_ or in CM_C2C12_, we do not observe a significant change in the cell morphology (data not shown) nor in cell cycle profile (Figure 4C). There were no changes in the cell morphology of B16 cells grown for 3 days in fibroblast conditioned media (Figure 4B, upper panels) and the percentage of apoptotic B16 melanoma cells did not vary significantly between 1 day or 3 days of exposure to CM_10T1/2_ (Figure 4B and C). In contrast, B16 melanoma cells incubated in (CM_C2C12_) for 3 days appear refractile and rounded (Figure 4B, upper panels). In addition, the cell cycle profiles show a marked increase in the percentage of apoptotic cells (40,5% versus 59,5% of cycling cells) (Figure 4B, C). The apoptotic effect of muscle conditioned media was not restricted to the B16 melanoma cells as we obtained similar results with Lewis-Lung carcinoma cells (Figure S2) indicating a specific effect by muscle conditioned media upon at least 2 metastatic cell types. As expected, CM_10T1/2_ did not have any apoptotic effect on the Lewis-Lung carcinoma cells (Figure S2).

### Muscle Conditioned Media Induces Reversible Changes in B16 Melanoma Cells

**Figure 5:**
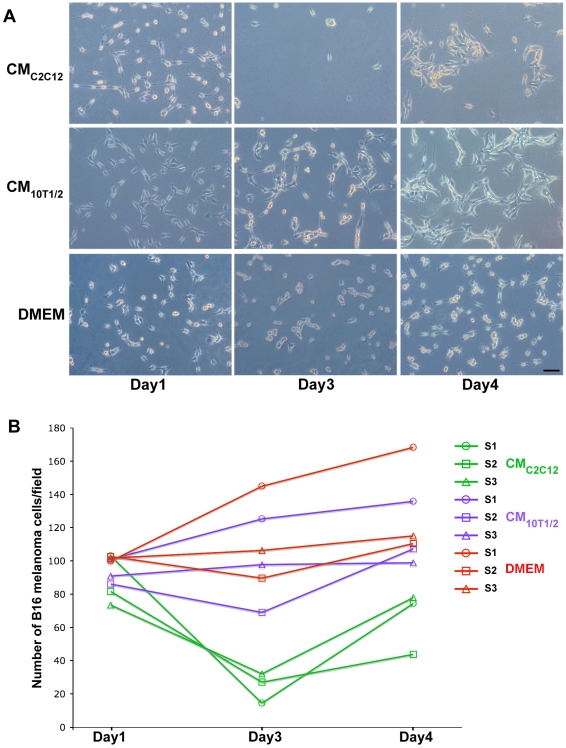
Emergence of reversibly resistant B16 melanoma colonies in CM_C2C12_ during a multiple selection process. A) Representative photomicrographs of B16 melanoma cells grown in CM_C2C12_, CM_10T1/2_, or serum free media (DMEM). CM_C2C12_ induces pronounced cell death by 3 days. However, 4 days after selection colonies of B16 melanoma cells start to appear. CM_10T1/2_ and DMEM do not affect the number of melanoma cells. Scale bar  = 40 µm. B) Schematic representation of the number of B16F10 melanoma cells/field after 1, 3 and 4 days of 3 successive rounds of selection in CM_C2C12_(green), CM_10T1/2_ (violet) and DMEM (red lines). In the first round of selection (S1) the number of melanoma cells grown in CM_C2C12_ (green line- circle) sharply decreases at Day 3. Cells grown in CM_10T1/2_ (violet) or in serum free DMEM (red) continue to proliferate. At 4 day the number of cells grown in CM_C2C12_ reaches 76% of the original number of cells at time of selection. The same trend is observed for the subsequent rounds of selection (S2,S3). For each time point, values represent a mean of 3 independent experiments. S1: Selection1; S2: Selection2; S3: Selection3. CM_10T1/2_ (violet lines) (*; p = 0.013). DMEM (red lines) (**; p = 0.006).

To assess whether metastatic cells acquire resistance to the muscle-mediated effects, we performed tumor suppression assays in which B16 cells were grown for prolonged periods in muscle conditioned media. Such assays typically result in the killing of most of the cells followed by the growth of resistant colonies. B16 cells were grown in the presence of CM_C2C12_, which induced pronounced cell death by 3 days and we observe that by 4–7 days, colonies of B16 melanoma cells appear. These colonies were allowed to recover in normal media and then subjected to two additional rounds of selection under identical conditions. The number of surviving cells for each selection cycle was determined. A pronounced decrease (83%) in the number of B16 melanoma cells was observed during the first round of selection with CM_C2C12_ (S1), 71% for the second round (S2) and 62% for the third round (S3) (Figure 5A,B, green lines). An average of 10 to 15 cells/field were observed after 3 days of selection in CM_C2C12_ (Figure 5A,B). By day 4, cells start to proliferate again forming small colonies (Figure 5A,B). Indeed, after 4 days of selection, the number of B16 melanoma cells that had resumed growth increased and reached 76% of the original number of cells at the time of selection. We conclude that while the B16 cells are growth suppressed in the presence of CM_C2C12_, they do not acquire a permanent change in cell behavior after multiple rounds of selection. B16 cells grown in (CM_10T1/2_) or DMEM alone continue to proliferate and the number of cells increases on average by 23% and 29% (Figure 5A,B) respectively during the 3 cycles of selection, confirming the absence of an apoptotic effect of serum free CM_10T1/2_ or DMEM on the B16 melanoma cells. Furthermore, these results point to a specificity of the muscle conditioned media on tumorigenic cells. After 3 days of selection, a statistically significant difference was observed in the proliferation rate of cells grown in CM_C2C12_ as compared to cells grown in either CM_10T1/2_ (p = 0.013) or serum free DMEM (p = 0.006). In contrast, there was no significant difference in the proliferation rate of melanoma cell grown in serum free DMEM as compared to melanoma cells grown in CM_10T1/2_. Selected B16 melanoma cells transferred to serum containing growth media (GM) resume normal behavior, including melanin secretion, further confirming that the inhibitory effects exerted by muscle cells upon B16 cells is completely reversible.

### Myogenic Cells Exert Density Dependent Growth Inhibition and Induce MiTF Downregulation on Parental and Selected B16-GFP Melanoma Cells

**Figure 6:**
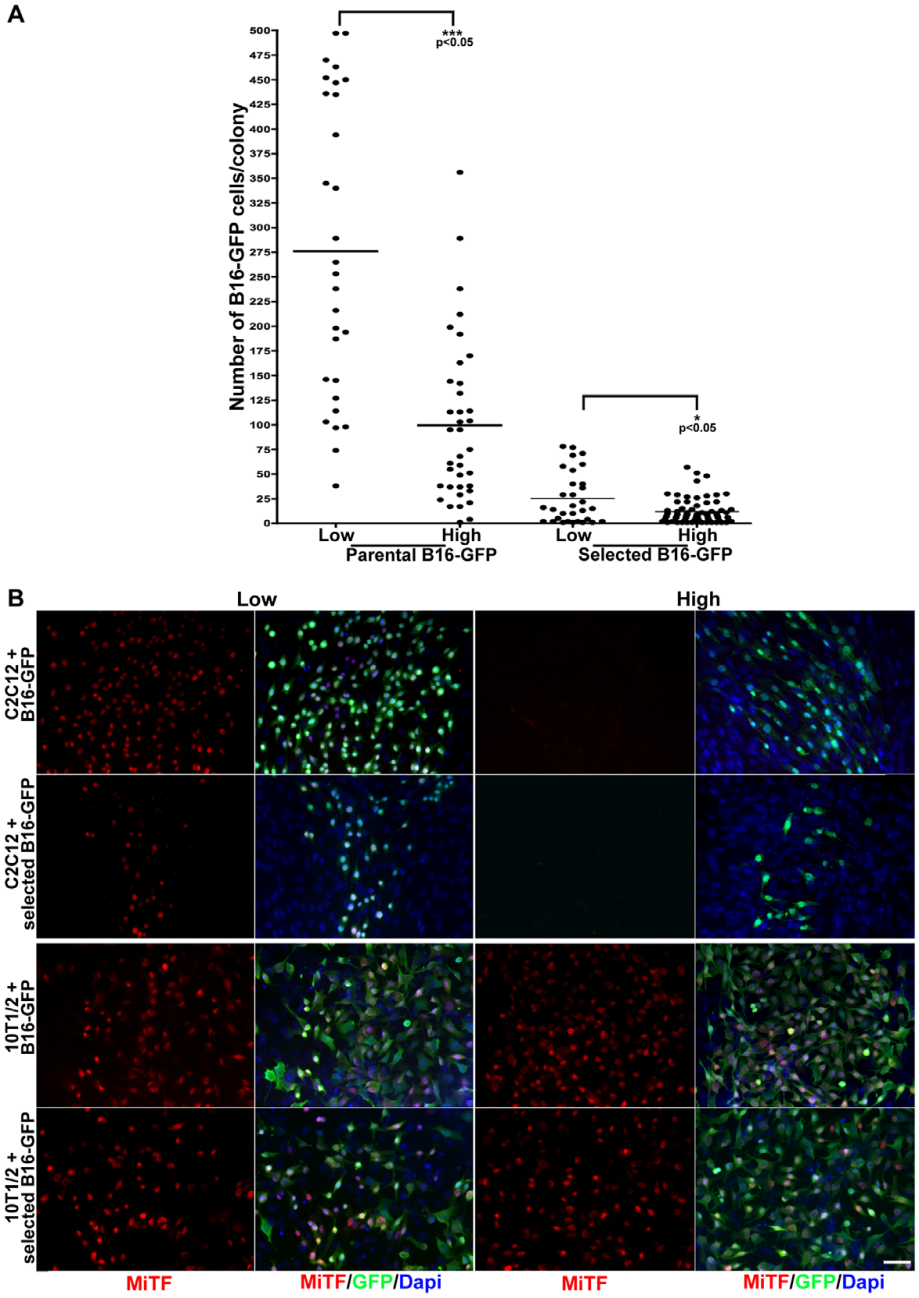
Myogenic cells exert density dependant growth inhibition and induce MiTF downregulation on parental and selected B16-GFP melanoma cells. A) Distribution of the number of cells per colony of either parental or selected B16-GFP melanoma cells, grown with C2C12 cells plated at low and high density. Muscle cells inhibit growth of melanoma cells. Cells previously exposed to muscle conditioned media are more sensitive to this effect. The inhibition is dependent on the density of muscle cell. The black lines represent the mean values of 3 independent experiments: 276 cells/colony for parental B16-GFP in low-density C2C12; 99 cells/colony for parental B16-GFP in high-density C2C12; 25 cells/colony for selected B16-GFP in low-density C2C12; 12 cells/colony for selected B16-GFP in high-density C2C12. B) Left Panels: parental (B16-GFP, green) or selected (selected B16- GFP, green) melanoma cells expressing GFP grown with low density C2C12 or 10T1/2 fibroblasts and immunostained for MiTF (red). MiTF is expressed in parental and selected B16-GFP cells. Fewer B16-GFP cells are present in cultures with C2C12 cells. Nuclei were visualized by Dapi staining (blue). Right panels: B16-GFP and selected B16-GFP melanoma cells grown with high density C2C12 or 10T1/2 fibroblasts and immunostained for MiTF (red). MiTF expression is completely abrogated in parental and selected melanoma cells cultured with C2C12 cells plated at high density. This effect is specific to muscle cells as it is not observed in high density 10T1/2 fibroblasts/B16-GFP co-cultures. Nuclei were counterstained by Dapi (blue). Scale bar  = 50 µm.

To analyze the contribution of cell-cell interactions to the muscle-mediated inhibitory effect upon metastatic cells, parental B16-GFP cells or B16-GFP that had undergone selection for 3 days in CM_C2C12_ were grown with C2C12 myogenic cells. C2C12 cells were seeded at 2 different densities (low and high) and parental or selected B16-GFP were plated at a ratio of 1/400 allowing for a clonal distribution of the melanoma cells and the number of B16-GFP cells per colony was counted on day 4. High density of muscle cell cultures results in a sharp decrease in the number of parental B16-GFP cells. As shown in Figure 6A, the mean value of 276 cells/colony obtained with parental B16-GFP cells in co-culture with C2C12 cells plated at low density significantly decreases to 99 cells/colony in co-cultures with high-density C2C12 myogenic cells (Figure 6A, p<0.05). Selected B16-GFP cells showed significantly lower mean values of 25 and 12 cells/colony in low and high-density C2C12 co-cultures respectively (Figure 6A, p<0.05). These results demonstrate a density-dependant inhibitory effect of myogenic cells upon melanoma cell growth. Furthermore, the inhibitory effect of the myogenic cells on the selected B16 melanoma cells is stronger than that of the parental cells under similar conditions (25 versus 276 cells/colony at low density C2C12; 12 versus 99 cells/colony at high density C2C12. Figure 6A). In all cases melanin production was not observed.

MiTF is a transcription factor required for melanocyte lineage survival and melanin production; MiTF is also implicated in melanoma progression and metastasis [52], [53], [54]. Analyses of MiTF reveal expression in both parental and selected B16-GFP cells grown with low density C2C12 as well as low density 10T1/2 fibroblasts (Figure 6B, left panels). In contrast, MiTF expression is not detectable in parental and selected melanoma cells cultured with C2C12 cells plated at high density (Figure 6B, top right panels). This effect is specific to muscle cells, as it is not observed in 10T1/2 fibroblasts/B16-GFP co-cultures (Figure 6B, lower right panels). Taken together, these data suggest that the inhibitory effect of muscle cells upon melanoma cells is density-dependent and targets MiTF expression.

## Discussion

The rarity of secondary metatastasis in skeletal muscle has provoked a number of investigations and hypotheses for over 100 years. One simple explanation for the apparent resistance of skeletal muscle to secondary tumors was proposed by Ewing [55] which suggests that the probability for the deposition of blood-born tumor cells reflects circulatory patterns and hemodynamics. Consistent with this view, we find that B16-F10 cells have a preferential distribution in the lung following tail vein injection (and see [31], [32]). However, while we observe that 100% of tumor cell-injected mice developed lung tumors, and 30–50% showed tumors in the liver, spleen and intestine, we consistently find no tumor appearance in skeletal muscle. Given the high number of cells injected into the circulation exceeding the number of circulating metastatic cells in a metastatic incident, mechanical models only provide a partial explanation. Results presented here lend support to the “seed and soil” hypothesis proposed by S. Paget (1889) in which specific interactions between the tumor cell and host environment favor or inhibit growth and metastasis [17]. In addition to our findings, it has been demonstrated that B16-F10 cells have a preferential cell adhesion to lung tissue [56], [57]. Furthermore, organ-selectivity is maintained in ectopically grafted tissues. Dunn Osteosarcoma is known to favor the lung as a target for metastasis and will target ectopic (subcutaneous) lung grafts whereas subcutaneous skeletal muscle is not targeted [58]. We observed that intra-peritoneal injection of the B16-F10 cells led to a preferential tumor growth in the intestine, suggesting direct dissemination of tumor cells. A similar distribution of tumor cells is observed when we injected the B16-F0 (versus the B16-F10) melanoma cell line with a wider metastatic range [32]. We found that tumor growths were evident in the bone but not in surrounding skeletal muscle despite close proximity of the two tissues. It has been amply demonstrated that many stem cells are able to target skeletal muscle consistent with there being no mechanical barrier in skeletal muscle to circulating cells [59], [60]. We note that for the B16-F0 and F10 melanoma lines, a high incidence of tumor growth is found in the heart (75%) indicating that the tumor suppressive properties of skeletal muscle are not shared by cardiac muscle. Taken together, we propose skeletal muscle has a local tumor suppressive effect.

B16-F10 cells display two distinctive features in response to co-culturing with myogenic cells: the absence of melanin production and the acquisition of a myotube-like morphology. It was reported that the presence of cutaneous hypopigmentation favorably influences the prognosis and survival rate of patients with malignant melanoma [61], [62]. In vitro, a correlation between expression of the secreted factor MIA (a key molecule involved in progression and metastasis of malignant melanoma) and decreased melanogenesis was shown in the human melanoma cell line HMB2 [53], [63]. However, similar invasive and migratory characteristics were found when comparing low or high melanin producing B16 melanoma sublines suggesting that melanin production and invasive behavior may not be tightly linked [64]. In our study, the inhibition of melanin production in the B16 melanoma cells in co-culture with myogenic cells could reflect a lower degree of differentiation and thus a higher predisposition or plasticity allowing for myogenic conversion of the melanoma cells. Staining of the myotube-like elongated B16-GFP cells with myogenic markers (MyoD and MF20) confirmed the fusion and incorporation of melanoma cells into myotubes. About 10% of myotubes in our co-cultures contained fused melanoma cells as evidenced by the presence of GFP suggesting that only a subpopulation of the melanoma cells undergo conversion. A similar frequency of GFP+ myotubes was observed in co-cultures of myogenic cells with the carcinoma cell line (LLC1) suggesting a general mechanism whereby myogenic cells inhibit expansion of metastatic tumors in skeletal muscle. Furthermore, upon fusion with human myogenic cells (CHQ), the B16-GFP cells express early murine myogenic markers such as Desmin suggesting the reprogramming and conversion of melanoma cells into myogenic cells. The absence of murine MyoD transcripts indicates that the conversion is probably driven by myogenic transcription factors from neighboring human nuclei within the same cytoplasm. In addition, B16-GFP cells were able to fuse with the nascent or the pre-existing myofibers when directly or intra-arterially injected into injured or uninjured TA muscle. Taken together, these data suggest that a subpopulation of B16 cells have stem-cell properties with enhanced phenotypic plasticity allowing their fusion and conversion to the myogenic lineage. Emerging evidence indicates that tumor cells contain a subpopulation of cells showing embryonic-like plasticity that correlates with invasive capacity [65], [66], [67], [68], [69], [70]. Several studies demonstrated that melanoma cells differentiate into endothelial-like cells leading to enhanced tumor growth [68], [71], [72].

Our observations suggest that the skeletal muscle microenvironment contributes to the partial loss of tumorigenicity through a phenotypic conversion into muscle. A similar reprogramming of multipotent melanoma cells was observed following their transplantation into embryonic neural crest [73], [74]. Specifically, when melanoma cells are placed into the embryonic neural crest, they followed the migratory pathways of surrounding crest cells and did not form tumors [73], [74]. It is possible that the melanoma cells that undergo phenotypic conversion into muscle possess stem cell characteristics. Interestingly, the stem cell marker, CD133 is found to be expressed in many different cancer types including human melanomas and is believed to be a characteristic of a cancer stem cell [75], [76], [77], [78], [79]. Furthermore, when delivered through the vascular network, mesenchimal stem cells and CD133+ cells can cross the endothelial barrier and will form muscle suggesting shared properties with metastatic cells [60], [80]. Adhesion molecules such as NCAM are expressed in melanoma cells as well as in muscle satellite cells, which may facilitate tumor-myoblast fusion [81], [82], [83]. Finally, the Pax3 transcription factor is expressed in both myogenic and melanoma cells and plays a key role in cell migration during embryonic development and specification of the muscle stem cell niche [84], [85]. Taken together, we propose that metastatic cells and migratory stem cells share an overlapping set of cellular characteristics which predisposes metastatic cells to respond to tissue-specific local clues that act upon cell fate. In this context, skeletal muscle exerts a dominant effect upon metastatic cells, recruiting them into the myogenic program resulting in the observed rarity of metastatic tumors in skeletal muscle of humans [11], [12], [13], [14].

The effect of muscle cells upon metastatic cells can be explained in part by the presence of diffusible signals secreted by muscle into the local milieu that act specifically on metastatic cells and not on non-metastatic cells. Muscle conditioned media exerts a specific apoptotic effect upon metastatic cells with pronounced melanoma cell death after 3 days, whereas no effect was observed upon non metastatic cells. Previous investigations have identified several muscle-produced factors capable of inhibiting metastatic growth including adenosine and unidentified low molecular weight factors acting through the A3 adenosine receptor [29], [30], however, adenosine mediated inhibition of cell growth was also found in non-metastatic cells [86], [87], [88]. In our study, we find that conditioned media from muscle had no effect on proliferation and death of normal cells revealing a specificity for metastatic cells and potential promise as an avenue of research to identify therapeutic approaches to metastatic cancers.

While a single factor may underlie the muscle-mediated effects on metastatic cells that we report here, our data support a mechanism whereby muscle exerts 3 specific inhibitory effects upon metastatic cells which are 1) cellular recruitment into the myogenic lineage, 2) a paracrine-mediated cytostatic and 3) paracrine-mediated cytotoxic response. It is well established that prolonged exposure of tumor cells to cytostatic/cytotoxic stimuli lead to selection of genetically resistant clones [89], [90], [91]. Clonal selection of resistant metastatic cancer cells constitutes a major problem for clinical treatments in which therapeutic options become exhausted over time. If such a process occurs in muscle, we would predict the eventual appearance of tumors in skeletal muscle. When we performed tumor suppressor assays in which B16 cells are grown for prolonged and repeated rounds in muscle conditioned media, about 20% of the cells ultimately resume growth but do not acquire permanent changes in behavior. Consistent with this proposal, B16 cells display a marked down-regulation of MiTF which is dependent upon cell density and cell-cell contact. A similar observation was made in a porcine model of spontaneously regressing melanoma in which MiTF was noted to be significantly downregulated during the regressive phase of the melanoma and upregulated during the progressive phase suggesting a central role for this protein in melanoma biology [92]. MiTF also controls the expression of Tbx2 and CDK2, which are involved in melanoma proliferation and suppression of senescence [93], [94], [95], in addition to anti-apoptotic genes such as Bcl2 and ML-IAP [96], [97]. Taken together, we conclude that conditioned media-mediated apoptosis and contact-dependant growth inhibition are two key mechanisms accounting for the effect of skeletal muscle on metastatic cells. The fusion and the subsequent myogenic reprogramming observed in vitro and in vivo, remain limited and specific to a subpopulation of B16 melanoma cells and is probably related to enhanced phenotypic plasticity. Finally, downregulation of MiTF and subsequent induction of apoptosis and inhibition of cell growth may underlie the limited expansion of melanoma cells when grown in a skeletal muscle microenvironment. Defining the muscle mediated signaling pathways that lead to MiTF downregulation will be of key importance in the identification of the factors responsible for muscle mediated tumor growth inhibition.

## Materials and Methods

### Ethics Statement

All work with mice was carried out in adherence to French Government guidelines as well as those of the NIH and was approved by the Pierre et Marie Curie University.

### Mice and *In Vivo* Experiments

Experiments were performed in 8 to 12 weeks old female C57BL/6J mice (Janvier, France). For intravenous (tail vein), intra-arterial (Text S1) and intraperitoneal injections, 5×10^5^ B16-F10-GFP or B16-F0-GFP cells were injected in 200 µl of PBS. Muscles and other organs were harvested 3 weeks after injection, analyzed for the presence of B16 melanoma cells and snap frozen in liquid nitrogen. Skeletal muscle regeneration was induced by a freeze-crush injury of the left *tibialis anterior* (TA), as previously described [98]. 24 hours post-injury, B16-F10-GFP melanoma cells (1×10^3^ cells in 30 µl PBS) were injected into the left injured and the right uninjured TA muscles. Control TA muscles were injected with PBS.

### Cell Culture

C2C12 myoblasts, B16-F10 and B16-F0 melanoma cells, Luis Lung Carcinoma (LLC1), BNL CL.2 liver cells and 10T1/2 fibroblasts were grown in DMEM supplemented with 20% Fetal bovine serum (Hyclone) (GM). Primary mouse myoblasts were obtained by enzymatic digestion of 18 days old embryos hind limb muscles as previously described [99], [100]. CHQ human muscle cells were a kind gift of Dr. Butler-Browne [101].

B16-F10 melanoma cells and Luis Lung Carcinoma (LLC1) were stably infected with a MoMuLV retroviral vector expressing the GFP protein under the control of the CMV promoter. Cells were sorted by Flow Cytometry to select for high GFP expression. For coculture experiments, B16-GFP or LLC1-GFP tumor cells were co-cultured with C2C12, 10T1/2, BNL CL.2, CHQ, at ratios of 1/5, 1/10, 1/5,1/100, 1/400, 1/500 (tumor cells/non-tumor cells) in GM. A ratio of 1/100 was used for all subsequent experiments. To induce myogenic differentiation, after 3 days in GM cells were switched to DMEM containing 2% (v/v) horse serum (GIBCO) (Differentiation Medium, DM) for 5 to 9 days. Primary mouse myoblasts were co-cultured with B16-GFP tumor cells in a 1/500 ratio (tumor cells/myoblasts) for 3 days in GM, and switched to DM for 4 days. For colony growth experiments and MITF detection C2C12 or 10T1/2 cells were co-cultured with B16-GFP tumor cells at a 1/400 ratio (tumor cells/non-tumor cells) for 2 days in GM, and switched to DM for 2 days.

### Immunostaining

Cells grown on 6,12 or 24 well plates and cryosections of mouse hind limb muscles were fixed in 4% paraformaldehyde and stained with antibodies against sarcomeric Myosin (MF20, Developmental Hybridoma Bank of the University of Iowa), MyoD (Santa Cruz), Laminin (Sigma), GFP (BD Pharmingen), and MiTF (Fisher Scientific). Antibody binding was visualized by using biotin-conjugated goat anti-mouse IgG followed by Cy3-conjugated streptavidin (Jackson Immunoresearch), and Alexa488 or Cy3 -conjugated conjugated goat anti-rabbit IgG (Molecular Probes). Nuclei were counterstained with DAPI (Sigma). Photomicrographs were obtained using a Leica inverted microscope (DMIL) a Leica confocal microscope (DM2500 TCS SPE) and a Leica DFC300FX camera. For detection of GFP+ myofibers, a narrow range of emission wavelength (511 to 532 nm) was used to avoid detection of autofluorescence [102].

### RT-PCR

Cells were collected and RNA was extracted using RNeasy minikit (Qiagen). RT was performed using SuperScriptII Reverse Transcriptase (Invitrogen). Murine specific primers to detect Desmin and MyoD transcripts were designed and a PCR was performed. PCR conditions were 94°C for 4 min, followed by 30 cycles of 94°C for 1 min, 62°C for 1 min, and 72°C for 1 min. Primers sequences used were as following: mDesF:5′GAGGTTGTCAGCGAGGCTAC3′; mDesR: 5′CGATGACTTGAGCTGGGTTC3′ mMyoDF: 5′AGTGTCCTGCAGGCTCAAAC3′; mMyoDR: 5′TCT GCT CTT CCCTTCCCTCT3′.

### Quantitative Analysis

Melanin production was quantified by determining the number of green cells showing black pigments of 10 randomly chosen fields in 3 independent experiments.

Myogenic conversion of B16-GFP and LLC1-GFP cells was quantified by determining the number of green myotubes expressing either MyoD or MF20, and expressing this as a percentage of the total number of myotubes per field (% of GFP+ myofibers). 10 randomly chosen fields in 3 independent experiments were analyzed.

For colony growth experiments, the number of GFP positive cells/colony was counted in triplicates. A total of 30 to 60 colonies were analyzed per experimental condition.

### Preparation and Collection of Culture Conditioned Medium

C2C12 or 10T1/2 cells grown to confluence in GM, were incubated in serum free DMEM (GIBCO) for 6,12,24,36 and 48 hours. At the end of the incubation period, the supernatant was collected, centrifuged, filtered through a 0.22 µm filter (Millipore), and frozen.

### Cell Cycle Analysis

Cells were cultured for 1 or 3 days in either CM_C2C12_ or CM_10T1/2_, washed with PBS, fixed with 1% paraformaldehyde and resuspended in PBS containing Propidium iodide (PI, 50 µg/ml, Molecular Probes), RNaseA (0.1 mg/ml, Sigma) and Triton X-100 (0.05%, Sigma). DNA content was analyzed by flow cytometry using a FACScan (Becton Dickinson, San Diego, CA). The percentage of cells with sub-G0-G1 DNA content was counted as apoptotic cells. Values represent the mean (± SEM) from 3 independent experiments.

### Tumor Suppression Assay

B16-F10-GFP cells were seeded in triplicates at a density of 12500 cells/cm^2^ in 60 cm Falcon culture dishes and grown in CM_C2C12,_ CM_10T1/2_, or in DMEM for 4 days. Media was changed once on day 3. After shifting them to GM for 2 days, cells were trypsinized and submitted to a new round of selection as described above. The selection process was repeated 3 times. The number of surviving cells was determined at day 1, 3, and for each selection round by counting the cells in 5 randomly chosen fields from 3 independent experiments. Statistical analysis was performed with Prism software using Student's unpaired t-test.

## Supporting Information

Text S1 Parlakian, et al. Material and methods.
(0.03 MB DOC)

Figure S1 Parlakian, et al. LLC1 carcinoma cells cultured with skeletal muscle cells participate to the myogenic program. A) Photomicrographs of GFP expressing Lewis Lung carcinoma cells (LLC1-green) grown alone or in co-culture with myogenic cells (C2C12), fibroblasts (10T1/2), or liver cells (BNL.CL2). We note the elongated morphology of LLC1 carcinoma cells when co-cultured with C2C12. Scale bar  = 100 µm. B) Representative photomicrographs of GFP expressing LLC1 cells (green) grown and differentiated in co-culture with C2C12 mouse myogenic cell line. GFP labeled carcinoma cells fuse with the C2C12 cells forming chimeric green myotubes, which are positive for myosin heavy chain (MF20, red) and the myogenic transcription factor MyoD (red). Nuclei were visualized by DAPI staining (blue). Scale bare =  15 µm.
(4.35 MB TIF)

Figure S2 Parlakian, et al. Apoptotic effect of the conditioned media from C2C12 muscle cells on LLC1 carcinoma cells. Phase contrast photomicrographs of LLC1 carcinoma cells cultured in serum free conditioned media from 10T1/2 fibroblasts (CM10T1/2) or in serum free conditioned media from muscle cells (CMC2C12) for 3 days. After 3 days in CMC2C12, LLC1 carcinoma cells are less numerous and appear rounded and clustered. Scale bar  = 50 µm.
(0.49 MB TIF)
