# Supplementary material for: Skeletal Muscle Phenotypically Converts and Selectively Inhibits Metastatic Cells in Mice
Source: PLoS One. 2010 Feb 18;5(2):e9299. doi: 10.1371/journal.pone.0009299 (PMC2823787; doi:10.1371/journal.pone.0009299)
Supplement: Text S1 — Parlakian, et al. Material and methods. (0.03 MB DOC) [file pone.0009299.s001.doc]

**Experimental procedures (supplemental)**

**Intra-arterial injection.**

The femoral artery and vein of both limbs was isolated at mid-thigh level under stereomicroscope. After clamping the femoral vein and two collaterals, a catheter was introduced in the femoral artery and 5x10^5^ B16-F10-GFP cells in 200µl of PBS or PBS were injected. Clamping was maintained for 10 min following the injection. Muscles were harvested 2 weeks after injection and snap frozen in liquid nitrogen for microscopic analysis.
