# Supplementary figures and images for: Skeletal Muscle Phenotypically Converts and Selectively Inhibits Metastatic Cells in Mice
Source: PLoS One. 2010 Feb 18;5(2):e9299. doi: 10.1371/journal.pone.0009299 (PMC2823787; doi:10.1371/journal.pone.0009299)

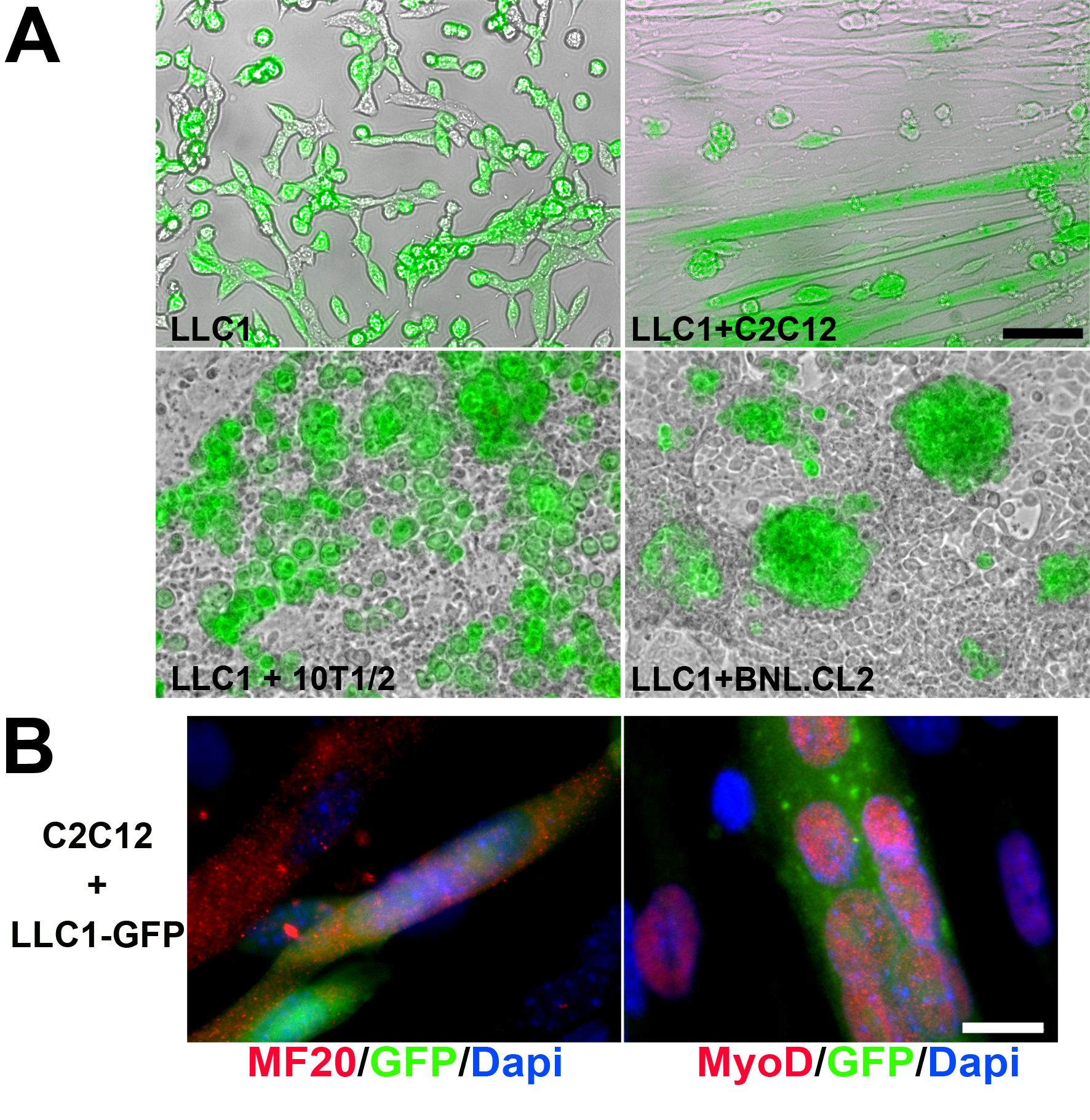

Supplement: Figure S1 — Parlakian, et al. LLC1 carcinoma cells cultured with skeletal muscle cells participate to the myogenic program. A) Photomicrographs of GFP expressing Lewis Lung carcinoma cells (LLC1-green) grown alone or in co-culture with myogenic cells (C2C12), fibroblasts (10T1/2), or liver cells (BNL.CL2). We note the elongated morphology of LLC1 carcinoma cells when co-cultured with C2C12. Scale bar = 100 µm. B) Representative photomicrographs of GFP expressing LLC1 cells (green) grown and differentiated in co-culture with C2C12 mouse myogenic cell line. GFP labeled carcinoma cells fuse with the C2C12 cells forming chimeric green myotubes, which are positive for myosin heavy chain (MF20, red) and the myogenic transcription factor MyoD (red). Nuclei were visualized by DAPI staining (blue). Scale bare = 15 µm. (4.35 MB TIF) [file pone.0009299.s002.tif]

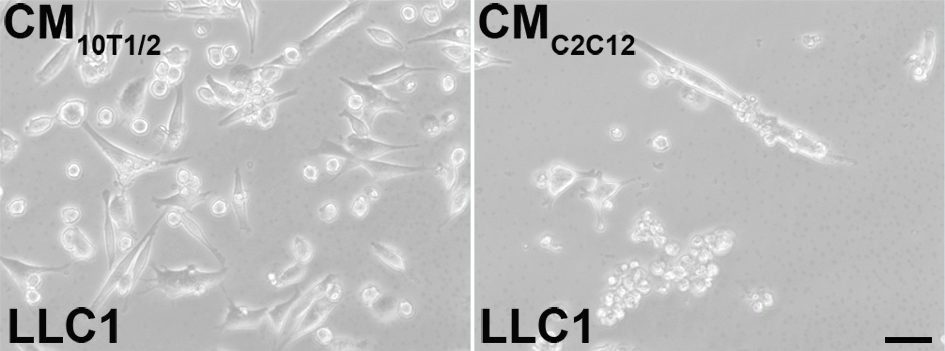

Supplement: Figure S2 — Parlakian, et al. Apoptotic effect of the conditioned media from C2C12 muscle cells on LLC1 carcinoma cells. Phase contrast photomicrographs of LLC1 carcinoma cells cultured in serum free conditioned media from 10T1/2 fibroblasts (CM10T1/2) or in serum free conditioned media from muscle cells (CMC2C12) for 3 days. After 3 days in CMC2C12, LLC1 carcinoma cells are less numerous and appear rounded and clustered. Scale bar = 50 µm. (0.49 MB TIF) [file pone.0009299.s003.tif]
